# Integrative analysis of outer membrane vesicles proteomics and whole-cell transcriptome analysis of eravacycline induced *Acinetobacter baumannii* strains

**DOI:** 10.1186/s12866-020-1722-1

**Published:** 2020-02-11

**Authors:** DineshKumar Kesavan, Aparna Vasudevan, Liang Wu, Jianguo Chen, Zhaoliang Su, Shengjun Wang, Huaxi Xu

**Affiliations:** 1 International Genomics Research Centre (IGRC), Jiangsu University, Zhenjiang, 212013 China; 2 Department of Immunology, School of Medicine, Jiangsu University, Zhenjiang, 212013 China; 3 Department of Laboratory Medicine, The Affiliated People’s Hospital, Jiangsu University, Zhenjiang, 212001 China

**Keywords:** *Acinetobacter baumannii*, Eravacycline, Outer membrane vesicles, Whole-cell transcriptome, OMVs proteome

## Abstract

**Background:**

*Acinetobacter baumannii* is a multidrug-resistant (MDR) hazardous bacterium with very high antimicrobial resistance profiles. Outer membrane vesicles (OMVs) help directly and/or indirectly towards antibiotic resistance in these organisms. The present study aims to look on the proteomic profile of OMV as well as on the bacterial transcriptome upon exposure and induction with eravacycline, a new synthetic fluorocycline. RNA sequencing analysis of whole-cell and LC-MS/MS proteomic profiling of OMV proteome abundance were done to identify the differential expression among the eravacycline-induced *A. baumannii* ATCC 19606 and *A. baumannii* clinical strain JU0126.

**Results:**

The differentially expressed genes from the RNA sequencing were analysed using R package and bioinformatics software and tools. Genes encoding drug efflux and membrane transport were upregulated among the DEGs from both ATCC 19606 and JU0126 strains. As evident with the induction of eravacycline resistance, ribosomal proteins were upregulated in both the strains in the transcriptome profiles and also resistance pumps, such as MFS, RND, MATE and ABC transporters. High expression of stress and survival proteins were predominant in the OMVs proteome with ribosomal proteins, chaperons, OMPs OmpA, Omp38 upregulated in ATCC 19606 strain and ribosomal proteins, toluene tolerance protein, siderophore receptor and peptidases in the JU0126 strain. The induction of resistance to eravacycline was supported by the presence of upregulation of ribosomal proteins, resistance-conferring factors and stress proteins in both the strains of *A. baumannii* ATCC 19606 and JU0126, with the whole-cell gene transcriptome towards both resistance and stress genes while the OMVs proteome enriched more with survival proteins.

**Conclusion:**

The induction of resistance to eravacycline in the strains were evident with the increased expression of ribosomal and transcription related genes/proteins. Apart from this resistance-conferring efflux pumps, outer membrane proteins and stress-related proteins were also an essential part of the upregulated DEGs. However, the expression profiles of OMVs proteome in the study was independent with respect to the whole-cell RNA expression profiles with low to no correlation. This indicates the possible role of OMVs to be more of back-up additional protection to the existing bacterial cell defence during the antibacterial stress.

## Background

Multidrug resistance (MDR) *Acinetobacter baumannii* is one of the most dangerous bacteria encountered among hospitalized and critically ill patients, particularly infecting the immunosuppressed patients, who undergo invasive procedures and are treated with broad-spectrum antibiotics [1]. Infections, such as ventilator-associated pneumonia (VAP), urinary tract infections, bacteraemia, complicated skin and soft tissue, abdominal and central nervous system infections are commonly caused by *A. baumannii* [2]. *A. baumannii* strains displaying MDR properties have increased significantly in the last decades [3]. *A. baumannii* species have an extensive capability of antimicrobial resistance in nature owing to their impermeable outer membranes and their environmental exposure to large reservoir of resistance genes [4]. The presence of wide range of resistance genes in *A. baumannii* succors easy evolution from the stress of antibiotics, making them extremely difficult in elimination. Some strains are also resistance to polymyxins—peptides making infected patient treatment more complicated and also impossible in some cases leading to fatality [5, 6].

Tigecycline is the first identified glycycyline antibiotic, belonging to the tetracycline class of antibiotics that is used as the last resort antibiotic for the treatment of MDR *A. baumannii* [7]. Eravacycline is a newer broad-spectrum synthetic fluorocycline with novel c-9 pyrrolidinoacetamido and c-7 fluoro modifications. Eravacycline is also successfully used against MDR strains in case of serious infections [8]. Reports have claimed that eravacycline showed broad-spectrum activity against most bacterial pathogens resistant with MIC_90_ values ranging from ≤0.008 to 2 μg/mL, except *P. aeruginosa* and *Burkholderia cenocepacia* (MIC_90_ values of 16–32 μg/mL) [9, 10]. In studies from the New York City Hospitals on 4000 contemporary Gram-negative pathogens, eravacycline MIC_50/90_ values (μg/mL) for *E. coli—K. pneumoniae*, *Enterobacter aerogenes*, *E. cloacae* and *A. baumannii* were 0.12/0.5, 0.25/1, 0.25/1, 0.5/1 and 0.5/1 respectively [11]. Eravacycline showed good activity against MDR strains expressing extended-spectrum β-lactamases, carbapenem resistance and other types of antibiotic resistance mechanisms in Enterobacteriaceae and *A. baumannii* [12].

MDR bacteria have developed mechanisms to combat antibiotic stress by changing a particular metabolic process. This has been a focus of research interest with many reports based on the expression analysis in bacterium upon antibiotic exposures [13]. Outer membrane vesicles (OMVs) are mainly seen among Gram-negative organisms helping them with cell to cell communication, secretion, pathogenesis, acquisition of nutrients, self-defence and antibiotic resistance [14]. The effective contribution of OMVs towards antibiotic resistance in bacteria, make it a very important tool in the research to combat drug resistance. To date; however, there are no elaborate studies in the area of proteomic analysis with a special focus on the proteins of the OMVs from *A. baumannii* upon non-natural eravacycline resistance induction. By studying the proteomic profile involved with OMVs, it could be possible to identify differential expressions of proteins which are related to response to antibiotic exposure. This can be further taken to the level of metabolic pathways involved with these proteins; thereby, possibly opening new avenues identifying drug targets or drugs. Yun et al., 2018 [15] had used a similar approach to study OMVs proteomics in imipenem treated clinical strain of *A. baumannii*. In our study, we used this resource to perform proteogenomic analysis of protein components of OMVs and RNA transcriptomics following eravacycline treatment. Similar reports on the in vitro antibiotic induced resistance and their expression profiles in *A. baumannii* are available with colistin [16] and meropenem [17]. However, for tetracycline group of antibiotics a similar induced resistance-based transcriptome profile and OMVs proteome analysis was not reported earlier to the best of our knowledge, except for one paper on the proteome analysis of *A. baumannii* DU202 strain under tetracycline stress [18]. Hence, with lack of prior transcriptome and proteome profiling of tetracycline drugs laboratory induced resistance, we focussed on the tetracycline group as they are the ones were newer drugs are being developed and some in pipeline for clinical usage. Eravacycline and tigecycline are promising drugs for MDR *A. baumannii* as they are relatively less affected by the common ribosomal protection proteins or efflux pumps [11] that usually confer resistance to tetracycline. In this present study we have done an integrative OMVs LC-MS/MS proteome analysis and whole-cell RNA sequence-based transcriptome analysis with both eravacycline induced (treated) and uninduced (control) *A. baumannii* strains.

## Results

### The evident increase in MIC of eravacycline upon induction of resistance

*Acinetobacter baumannii* strains were exposed to sequential passages of increasing concentration of eravacycline for evaluating the acquisition of resistance. The strains with acquired resistance were evaluated for gene expression profiling, and OMV proteome analysis was studied to identify a specific pattern in OMVs pertaining to resistance. The MICs of eravacycline for the *A. baumannii* ATCC 19606 and JU0126 strain were 0.125 and 0.5 μg/mL, respectively. The serial passage-based induction of acquired resistance among the isolates was carried out from 1/8th MIC (0.015625 μg/ml and 0.0625 μg/ml concentration for *A. baumannii* ATCC 19606 and JU0126 strain respectively) values and above, until a lethal concentration was reached above the MIC concentration. *A. baumannii* ATCC 19606 was able to resist the passages up to concentration of 64× MIC (8 μg/ml concentration), after which it failed to overcome the action of eravacycline. *A. baumannii* JU0126 strain tolerated upon induction till concentrations of 64× MIC (32 μg/ml concentration) of eravacycline, above which turned to be lethal concentration. Both organisms were able to present a resistant phenotype below 128× MIC concentration of eravacycline.

### High-throughput RNA sequence analysis

RNA sequence analysis was performed for the *A. baumannii* strains, ATCC 19606 and JU0126, respectively. Both strains were grown under 64× MIC concentrations of eravacycline obtained as per induced resistance protocol described above. From high-throughput RNA sequence analysis, the length of 16,205,012 (an error probability of 0.03%) and 15,029,752 (an error probability of 0.02%) clean reads were obtained from eravacycline resistance induced ATCC 19606 and JU0126 strains, respectively. A total of 18,732,924 (an error probability of 0.03%) and 16,680,372 (an error probability of 0.03%) clean reads were obtained from control untreated strains of ATCC 19606 and JU0126 strains, respectively. The Q20 of all these four samples reached 98% for treated and 97% for control which indicated a high quality of transcriptome sequencing. The GC content (%) were 44.03 and 43.87 (ATCC 19606 and JU0126, respectively) for treated strains and 45.11 and 45.04 (ATCC 19606 and JU0126, respectively) for control strains. Pearson’s correlation between each sample was analysed: ATCC 19606 control with ATCC 19606 treated strains, had a value of 0.774 and JU0126 control had 0.735 correlation values with treated JU0126. ATCC 19606 control strains had 0.848 correlation value with treated JU0126, ATCC 19606 control had 0.866 correlation value with control JU0126 and JU0126 control strain had 0.926 correlations with treated ATCC 19606 strains. These correlation values show high correlation between the samples.

### Significant DEGs among the eravacycline treated strain when compared with untreated control strains

**Fig. 1:**
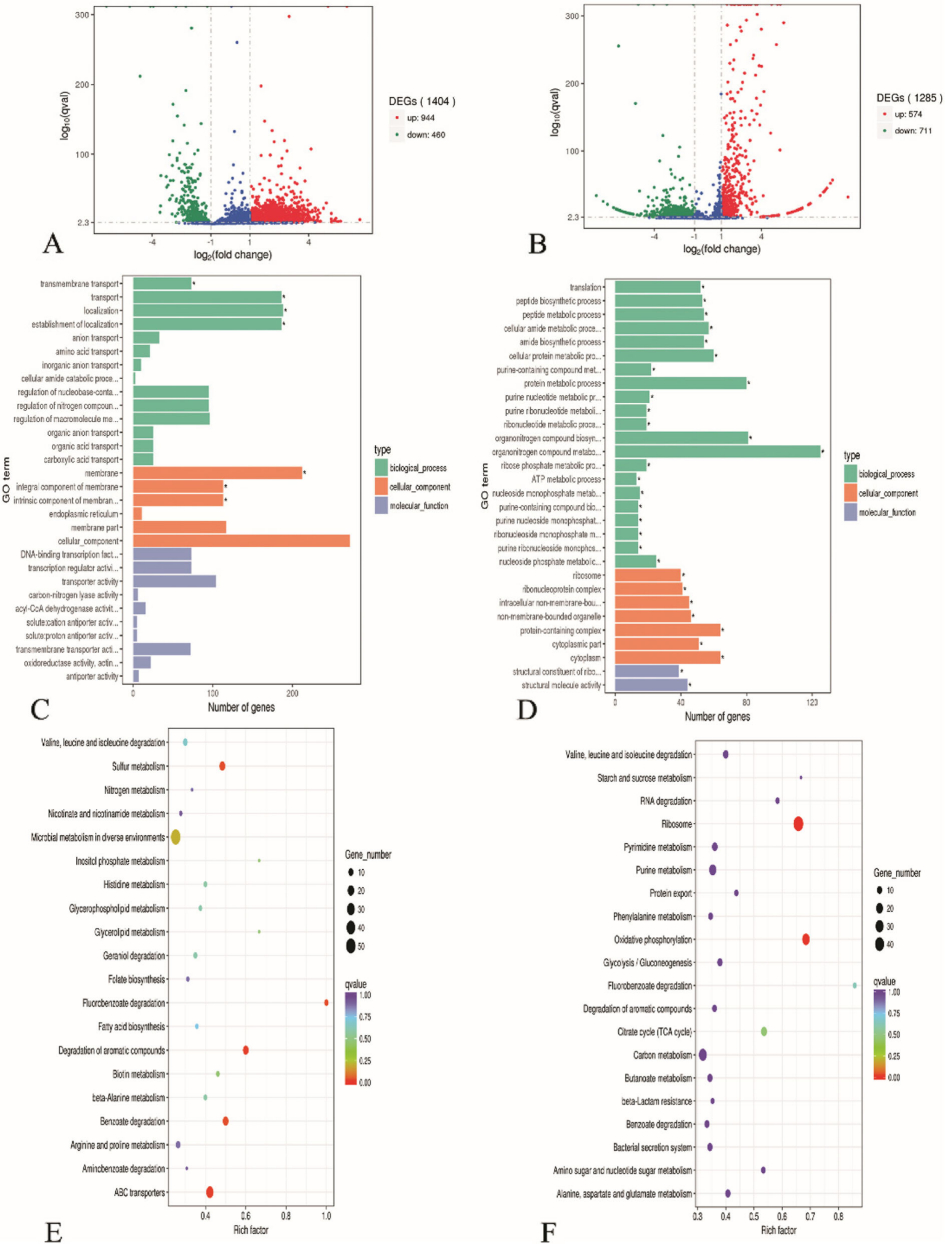
**a**, **b** Comparison of differentially expressed genes (DEGs) between eravacycline treated and control samples of *A. baumannii* ATCC 19606 and clinical strain JU0126. A volcano plot analysis was used to plot the DEGs between control and treated samples of ATCC and JU0126 strains of *A. baumannii*. Red dots represent upregulated DEGs, green dots represent downregulated DEGs and blue. dots represents no significant change between samples. **c**, **d** GO enrichment analysis of differentially expressed genes in eravacycline induced versus control *A. baumannii* ATCC 19606 and JU0126 strains. The DEGs were categorized into biological (green), cellular (red) and molecular function (blue) components. **e**, **f** Scatter plot representation of enriched KEGG pathway statistics of DEGs from *A. baumannii* ATCC 19606 and JU0126 strains

The complete gene expression values for *A. baumannii* ATCC 19606 and JU0126 eravacycline treated strains are provided in Additional file 2. In an effort to study the changes in the biological mechanisms and/or pathways of the bacterial system upon resistance to eravacycline in the treated strains when compared with the control eravacycline susceptible strains of *A. baumannii* ATCC 19606 and JU0126, DEGs analysis was performed (Additional file 3; Fig. 1a, b). For DEGs analysis, parameters *P* values (< 0.05) and fold changes ≥2 were used. A total of 944 DEGs (67.2%), 574 DEGs (44.7%) were upregulated and 460 DEGs (32.8%), 711 DEGs (55.4%) downregulated in *A. baumannii* ATCC 19606 and *A. baumannii* JU0126 respectively.

### GO enrichment analysis of DEGs

GO enrichment is widely used to find the biological roles of each gene and its products [19]. All DEGs were mapped to their terms in GO database and compared with the reference transcriptome. GO mapped DEGs from ATCC 19606 and JU0126 were identified and classified into functional groups in three main categories: biological process, cellular process and molecular function (Fig. 1c, d). Totally, 2219 and 2018 GO terms were identified in the DEGs from *A. baumannii* ATCC 19606 and JU0126 respectively under all three categories with localization, transport, cellular component, membrane, transport activity, and transmembrane transport activity being dominant terms. In JU0126, organonitrogen compound metabolism, protein metabolism, protein-containing complex, cytoplasm, structural activity and structural constituent of ribosome were dominant terms in all three categories, significantly enriched GO terms were considered based on the corrected *P* < 0.05.

### Kyoto encyclopedia of genes and genomes (KEGG) analysis of DEGs

KEGG database is a collection of various pathways, which represents the molecular interactions network between each gene/proteins [20]. To identify the enriched pathways involved in eravacycline induced strains of *A. baumannii* ATCC 19606 and clinical strain JU0126, KEGG analysis was done. In total, 78 and 86 pathways were identified in the DEGs of ATCC 19606 and JU0126 strains, respectively. The enriched factors were represented in the ratio of the differentially expressed gene number to the total gene number in a certain pathway. The values were represented in *Q* value, which is a corrected *P-*value ranging from 0 to 1. The size and color gradient of the dots indicate the range between *Q* value and the number of DEGs mapped to the indicated pathways, respectively. The top 20 values are shown in Fig. 1e and f.

### Quantitative reverse transcriptase-polymerase chain reaction (qRT-PCR) validation of DEGs

**Fig. 2:**
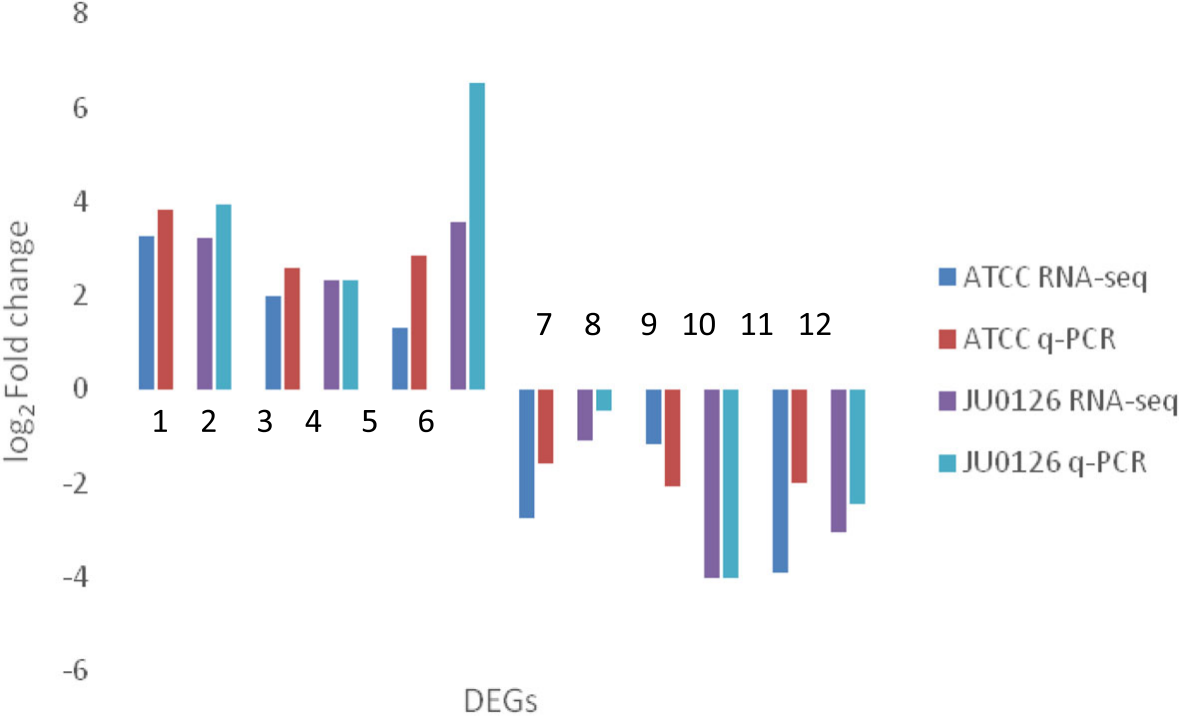
qRT-PCR analyses of six DEGs from each *A. baumannii* ATCC 19606 and JU0126 strain. Upregulated genes of ATCC 19606- 1: AUO97_00445, 3: AUO97_00560, 5: AUO97_00700. Downregulated genes of ATCC 19606-7: AUO97_05635, 9: AUO97_15195, 11: AUO97_11755. Upregulated genes of JU0126- 2: AUO97_02660, 4: AUO97_03195, 6: AUO97_00745. Downregulated genes of JU0126- 8: AUO97_11755, 10: AUO97_18615, 12: AUO97__18630

The genes for qPCR were chosen based on their involvement towards eravacycline/tetracycline resistance and also from the RNA sequence analysis, those genes which were highly up-regulated and also the most down-regulated ones, when compared to the untreated (uninduced) strains were validated by qPCR. Six DEGs from both upregulated and downregulated genes for both the strains were selected from the RNA sequence analysis and validated through qRT-PCR study (Fig. 2). From the ATCC 19606, the genes multidrug efflux RND transporter permease subunit (AUO97_00445), MSF transport (AUO97_00560), M1 family peptidase (AUO97_00700) which were upregulated with 3.2731, 1.9644, 1.2859 log_2_-fold respectively in RNA-sequencing data, showed 3.8204, 2.5822 and 2.8533 log_2_-fold changes respectively using the qRT-PCR analysis. From the RNA-sequencing data, genes porin (AUO97_05635), trifunctional transcriptional regulator (AUO97_15195) and transfer-RNA (AUO97_11755) which were downregulated with − 2.782, − 1.176 and − 3.9366 log_2_-fold respectively, displayed-1.5859, − 2.0788 and − 2.0203 log_2_-fold changes, respectively using the qRT-PCR analysis. From the JU0126 strain, the genes corresponding to Ade B pump (AUO97_02660), membrane protein (AUO97_03195), class C extended-spectrum β-lactamase ADC-26 (AUO97_00745) were upregulated with 3.2339, 2.3114 and 3.5588 log_2_-fold change, respectively in RNA-sequencing data, showed 3.9497, 2.3170 and 6.5163 log_2_-fold change, respectively, using the qRT-PCR analysis. The genes that downregulated in the RNA-sequencing data, transfer RNA (AUO97_11755), iron-containing alcohol dehydrogenase (AUO97_18615) and aldehyde dehydrogenase (AUO97__18630) with − 1.0926, − 4.0487 and − 3.0715 log_2_-fold change respectively, showed − 0.4585, − 4.0264 and − 2.464 log_2_-fold change using the qRT-PCR analyses, respectively.

### Transmission electron micrograph of OMVs

**Fig. 3:**
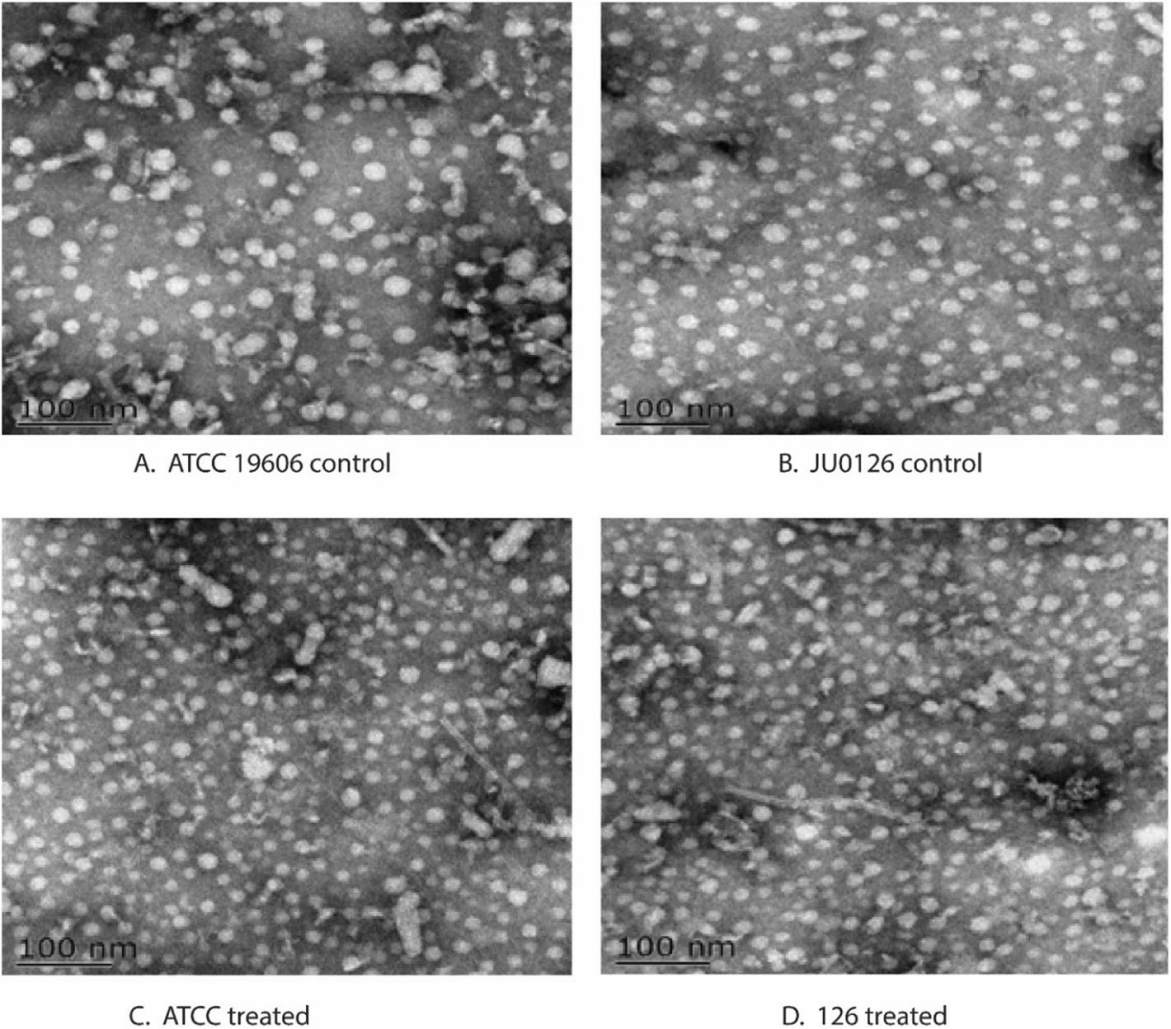
Transmission electron microscopic image of OMVs. **a** OMVs from *A. baumannii* ATCC 19606 control strain, **b** OMVs from *A. baumannii* ATCC 19606 strain treated with eravacycline, **c** OMVs from *A. baumannii* JU0126 control strain, **d** OMVs from *A. baumannii* JU0126strain treated with eravacycline

Transmission electron micrograph images from the negatively stained *A. baumannii* showed the presence of OMVs in both the ATCC 19606, JU0126 control and treated strains with an abundance of OMVs observed from the treated strains (Fig. 3a–d). It is known that OMVs are associated with bacterial survival, nutrient uptake, environmental stress and biofilms [21]; and, this is evident in the present study with an increased OMV presence in strains exposed to eravacycline induction.

### Effect of eravacycline induction on the OMV proteome

The OMVs proteome of eravacycline treated and control ATCC 19606 and clinical strain JU0126 were analysed using LC-MS/MS study which resulted in the identification of 227 and 342 proteins for control ATCC 19606 and treated, respectively. Similarly, 203 and 265 proteins were identified for the OMVs from JU0126 control and treated strains, respectively (Additional file 4). These proteins were analysed further using pSORTb v3.0.2 and SignalP v5.0. The occurrence of Omp38 and entericidin EcnA/B family proteins were of high intensity within the OMVs taken from the eravacycline treated clinical strain JU0126 based on the LC-MS/MS proteome analysis. Apart from the above two highly enriched proteins, a total of 10 other Omp proteins were also identified in the OMVs from eravacycline induced clinical strain JU0126. Overall, the resistance-associated proteins in OMVs identified from the proteome analysis of the eravacycline treated clinical strain JU0126 were: porin, outer membrane porin D, ABC transporter, substrate-binding protein family V, OmpA family protein, Omp38, Omp transport protein Ompp1, putative acriflavine resistance protein A, transcriptional regulators AraC and TetR family, major facilitator family protein and β-lactamase. The same OMP repeated to have high intensity in the OMVs from ATCC 19606 strain, and they are Omp38 and entericidin EcnA/B family proteins. Resistance proteins present in the OMVs from eravacycline treated ATCC 19606 strain, includes porin, Ompp1, OmpA family protein, OmpW, Omp38, Omp85, OprM efflux pump, OprD, GntR regulator and TetR regulator.

### Subcellular localization of proteins from OMVs

**Fig. 4:**
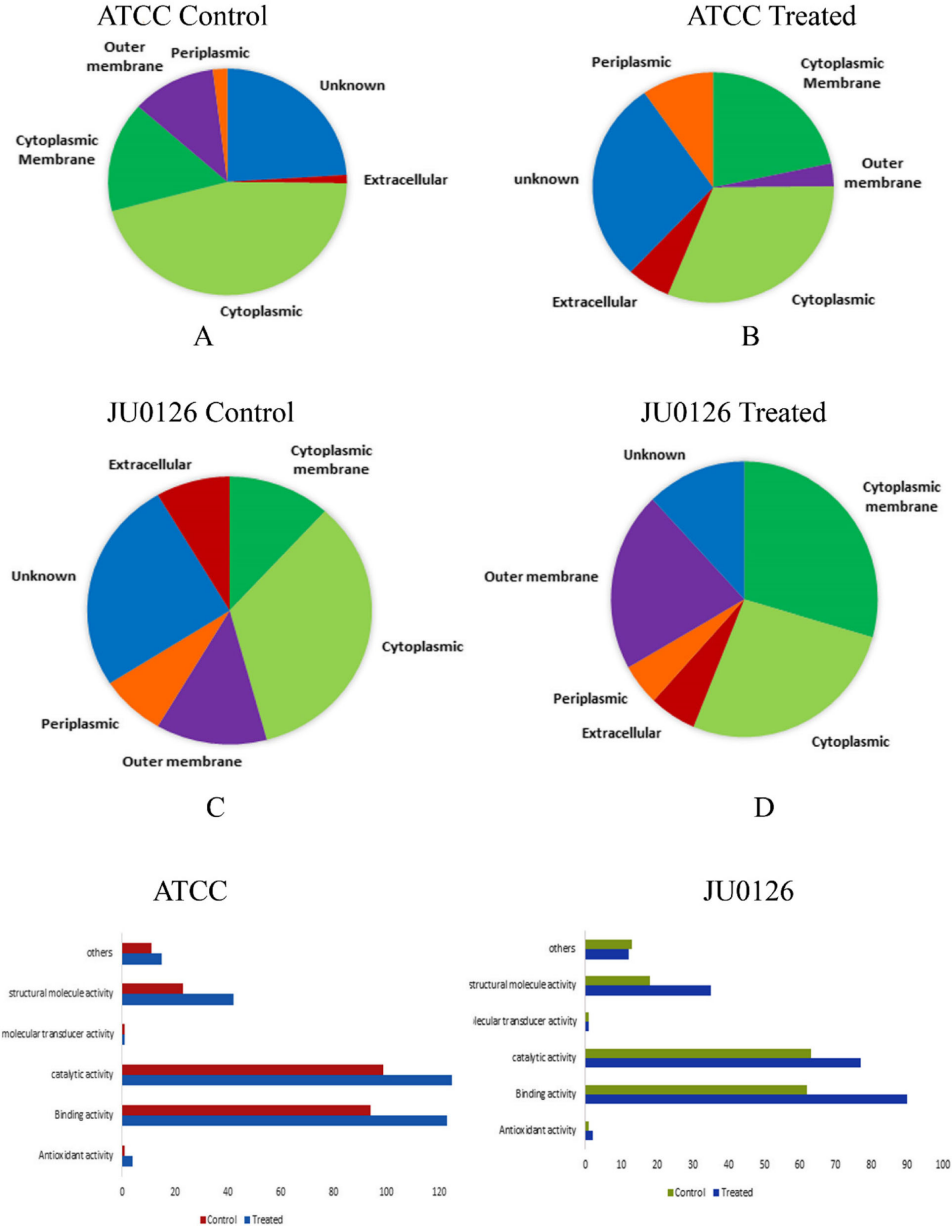
Classification of subcellular localization of proteins from OMVs of eravacycline control and treated strains of *A. baumannii* ATCC19606 (**a**, **b**) and JU0126 (**c**, **d**). Gene ontology annotations of OMVs proteins from control and eravacycline treated *A. baumannii* ATCC19606 (**e**) and JU0126 strains (**f**) using STRAP software

Figure 4a shows the subcellular localization of the 254 OMV proteins from the eravacycline-untreated control *A. baumannii* ATCC 19606 strain and the 342 OMV proteins from the eravacycline treated *A. baumannii* ATCC 19606 strain. The comparison of the pie distribution of the protein localization among the antibiotic-induced and uninduced strains showed the difference in the total number of proteins. In the *A. baumannii* ATCC 19606 strain (treated), eravacycline induction (Fig. 4b) resulted in the increase in proteins pertaining to different functions: cytoplasmic membrane proteins with antibiotic resistance functionality included outer membrane family proteins, outer membrane assembly complexes, OMP, OMP assembly factor, putative RND efflux pumps, carbapenem-associated resistance proteins, and OXA-51 family carbapenem-hydrolyzing class D β-lactamase OXA-98. Stress tolerance proteins, peptidases, transcription termination factors, and many ribosomal proteins were also localized in the cytoplasmic membrane. Antibiotic resistance-related proteins localized in the outer membrane, includes Metallo β-lactamases-fold metallohydrolase, OmpW family protein, outer membrane insertion signal domain protein, ABC transporter family protein, ompA family protein and along with ribosomal proteins. Cytoplasmic proteins expressed were OMP transport protein, Ompp1/FadL/TodX family, outer membrane efflux protein OprM, ATP-binding cassette protein along with ribosomal proteins, elongation factors, and transcriptional regulator.

Figure 4c and d shows the cellular localization of the 214 proteins from the OMVs isolated from the untreated control of clinical isolate *A. baumannii* JU0126 and 265 proteins from eravacycline treated *A. baumannii* JU0126 strain respectively.

### Functional annotation of proteins from OMVs

The annotation of the differentially expressed protein was done using the STRAP tool that uses an exhaustive database of Uniprot, EBI and GO to classify the proteins based on their biological process, cellular component and molecular function [22]. Figure 4e represents the proteins from the eravacycline treated *A. baumannii* ATCC 19606 and control strains associated with different functional terms. And the proteins annotated in the OMVs from the clinical strain *A. baumannii* JU0126 treated and control were classified based on their function as shown in Fig. 4f.

### Presence of enriched genes and proteins functioning as virulence factors and resistance determinants

**Fig. 5:**
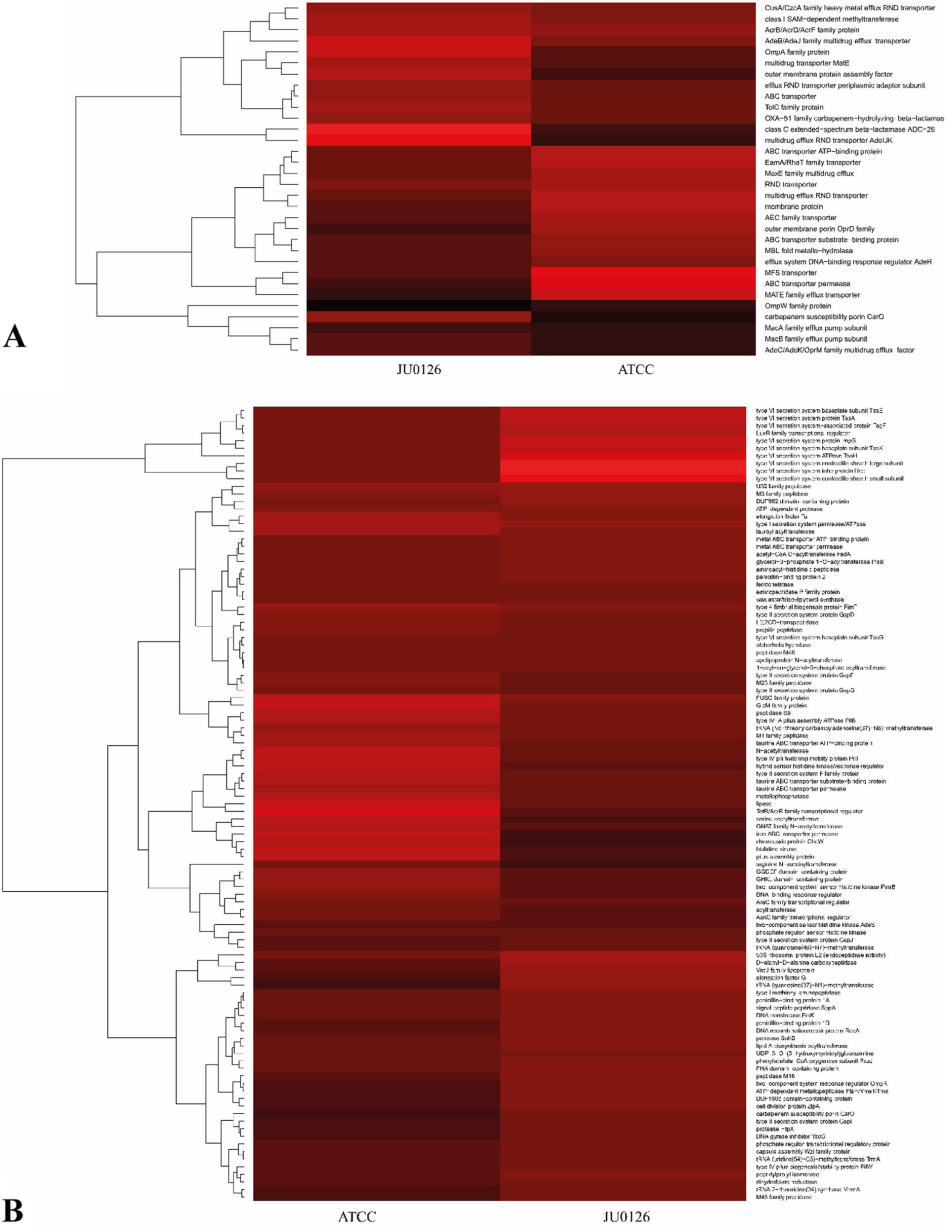
Distribution of genes pertaining to antibiotic resistance and virulence in ATCC 19606 and JU0126 *A. baumannii* strains. Each block of gradient colors, red (high) to black (low) represents the fold change expression of resistance (**a**) and virulence (**b**) genes from transcriptome analysis of ACC and JU0126 A. baumannii strains

The genes (functions pertaining to virulence, stress response and antibiotic resistance) expressed (from RNA sequencing) in the eravacycline induced *A. baumannii* strains of ATCC 19606 and JU0126 were compared with the uninduced controls with respect to their log_2_-fold change (Fig. 5a, b). *A. baumannii* has many innate virulence factors and resistance proteins, many of which have been described in detail by Lee et al. [23]. Protein secretion systems are among the major virulence factors in Gram-negative bacteria, they function by assisting in the process of transporting proteins between cellular locations [24]. Genes were considered as differentially expressed when the log_2_-fold change was > 2-fold.

**Fig. 6:**
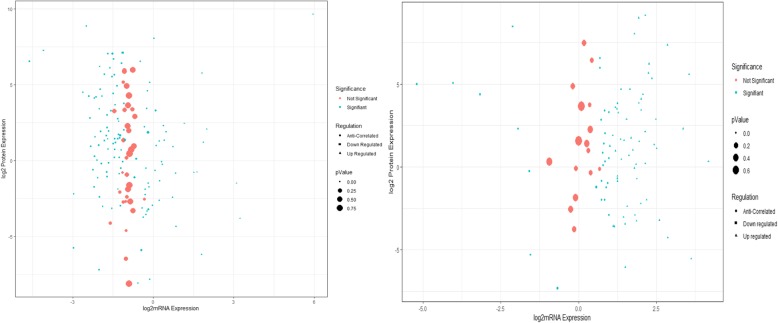
Comparison of whole-cell transcriptome and OMV proteome. **a***A. baumannii* ATCC 19606, **b***A. baumannii* JU0126. The log2-fold change represents the ratio of eravacycline treated: control condition. *p*-value less than 0.05 was considered significant. Genes with no protein expression are considered anticorrelated. Up- and downregulation of genes/proteins are designated depending on positive or negative log2-fold change, respectively

The mRNA expression data were compared with the protein abundance dataset based on their differential expression (Additional file 5). The correlation between mRNA expression and protein expression for all the genes from OMVs in both the treated and control strains of ATCC 19606 and JU0126 was represented with a correlation coefficient. Overall, comparing mRNA and protein expression from our data, there was a very low correlation (*r* = 0.0184 from ATCC 19606 and *r* = 0.0038 for JU0126). Figure 6a represents the correlation between whole-gene mRNA expression and OMV proteome based on both log_2_-fold change and *P-*value for the strain *A. baumannii* ATCC 19606 and Fig. 6b the same for JU0126 strain. In the strain *A. baumannii* JU0126, very few proteins displayed linear correlation with the similar expression pattern in whole-cell mRNA and OMV protein abundance; they are 30S ribosomal proteins S9, S3, S5, 50S ribosomal proteins L2, L16, L1, L18 and L28.

### DEGs/proteins belonging to the most highly enriched biological pathways

**Fig. 7:**
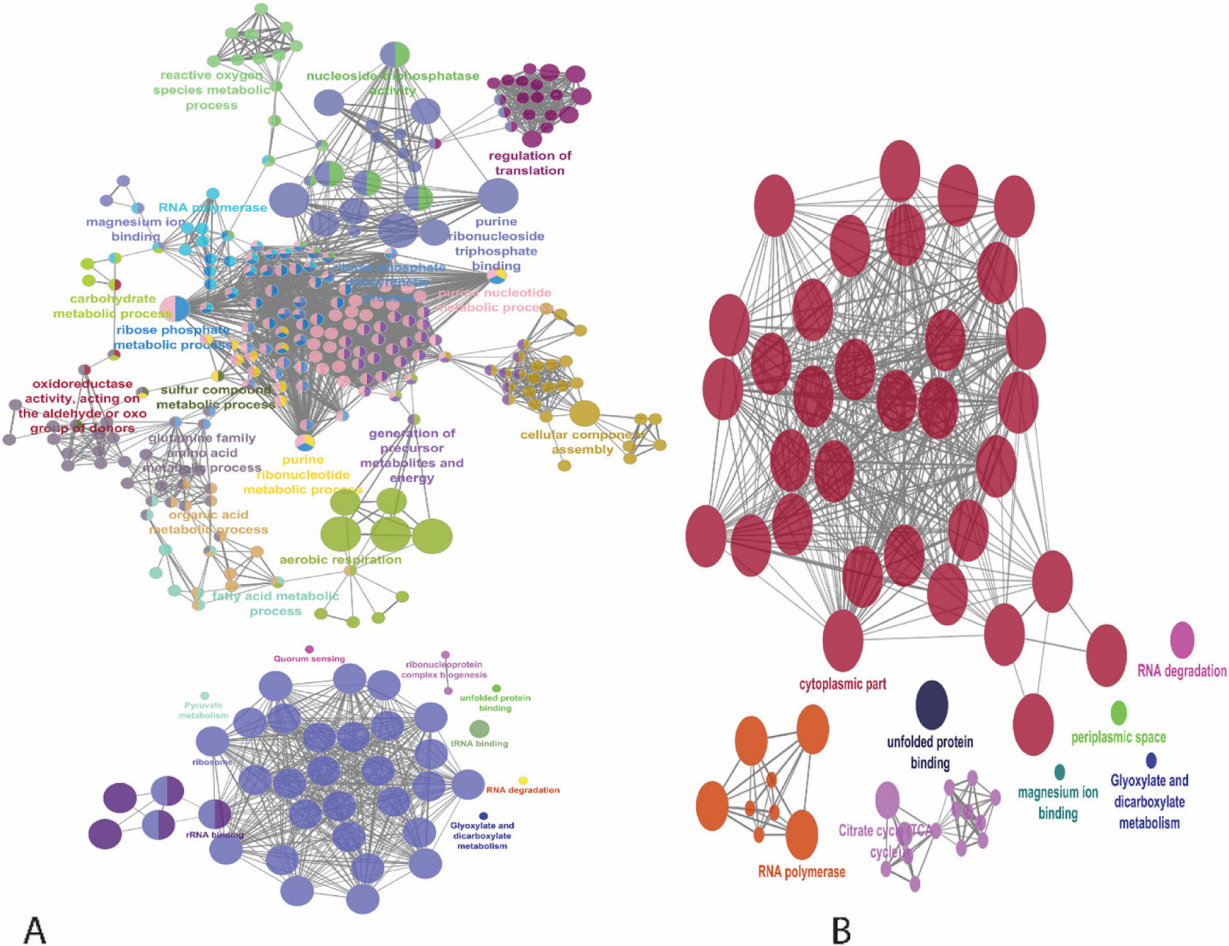
Go enrichment analysis and visualization of genes/proteins from both mRNA and OMVs proteome of *A. baumannii* ATCC 19606 (**a**) and JU0126 (**b**) strain using ClueGO/CluePedia plug-in from Cytoscape software. The node colors were represented to the biological, molecular and cellular functions of the genes/proteins according to the significant association of related GO terms

Two PPI networks were constructed using string database for ATCC 19606 and JU0126 strains. The commonly expressed gene/proteins from both transcriptome and OMVs proteome were selected and used to build the PPI network. For ATCC 19606, 328 nodes and 3603 edges and 83 nodes and 545 edges for JU0126 were generated from string database (Additional file 6). Both ATCC 19606 and JU0126 PPI networks were visualized using Cytoscape. The *P*-value of mRNA versus protein from both ATCC 19606 and JU0126 were used for node size and combine score for edge size generation in PPI network. Using ClueGO/CluePedia plug-in of Cytoscape software, enrichment pathways for commonly identified genes/proteins from both mRNA and OMVs proteome were analysed. For *A. baumannii* 19606 (Fig. 7a) high enrichment of biological processes belonging to “ribosome”, “RNA polymerase”, “regulation of translation”, “nucleoside phosphate”, “purine nucleotide metabolic process”, “rRNA binding” and “tRNA binding” were found in the functional analysis. In the functional analysis, high enrichment of processes pertaining “ribosome”,” ribosomal subunit” and “RNA polymerase” were identified in *A. baumannii* JU0126 (Fig. 7b).

Highly interactive and subgraph network was generated using the MCODE plug-in from Cytoscape software. For *A. baumannii* ATCC 19606, 14 efficient clusters and 4 for JU0126 strain were identified, for further analysis nodes with *n* > 10 clusters were selected from both strains (Additional file 7). Four clusters for ATCC 19606 were selected, the first cluster consisted of 54 nodes with a score of 51.32, the second, third and fourth clusters had 11, 18 and 11 nodes with scores 10.6, 9.8 and 7.2, respectively. Cluster one consisted majorly of ribosomal proteins, proteins for RNA polymerases, elongation factors, intracellular organelles, ribosomal subunits, tRNA binding and regulation of translation, cluster two included cell envelope organization and cluster three with response to toxic substance. For the strain JU0126, only one cluster was taken with 25 nodes and 23.3 scores, that included ribosomal subunit, RNA polymerase, cellular macromolecules biosynthesis and cellular nitrogen compound biosynthesis. The enlarged view of each cluster is represented in Additional file 8, figure A–D for ATCC 19606 and figure E for JU0126 respectively.

## Discussion

*A. baumannii* is known for its many intrinsic resistance determinants (present irrespective of an antibiotic exposure) that are often missed due to their low-level of resistance displayed phenotypically. However, upon induction mostly due to an antibiotic exposure these resistance genes are either over-expressed or under-expressed (as in case of porins) contributing to very high resistance. In the present study, tigecycline resistant clinical isolate *A. baumannii* strain JU0126 were induced in vitro for resistance to eravacycline. Whole-cell transcriptome analysis was performed for both eravacycline induced and non-induced strains of *A. baumannii* strains. In addition, OMVs were isolated from both strains and their proteomes studied from both eravacycline induced JU0126 strains. The whole-cell transcriptome expression was compared with OMVs proteome in JU0126 strains. To better understand the transcriptome profiles of the clinical isolate, an integrated analysis of the results was done with the transcriptome data from already sequenced eravacycline-susceptible quality control strain *A. baumannii* ATCC 19606 [25] in a similar experimental protocol to the clinical isolate*.* The *A. baumannii* ATCC 19606 strain was used as a reference for the study along with the clinical isolate and the comparative study was focused only between the expression profiles of the un-induced and the laboratory-induced eravacycline resistant phenotypes.

### Upregulated DEGs/proteins

Genes pertaining to the family of drug efflux and membrane transport were significantly high in expression among both ATCC 19606 and JU0126 strains. The genes that were upregulated in the eravacycline treated ATCC 19606 strain in comparison with untreated strains included majorly of efflux and transporter families. The multidrug efflux RND transporter permease subunit gene and major facilitator superfamily (MFS) transporter were significantly overexpressed in the ATCC 19606 treated strain. Although specifically, AdeB pump and some membrane proteins were upregulated in the eravacycline treated JU0126 strain. The eravacycline-based antibiotic induction in bacterial strains leading to the upregulation of MDR pumps can be supported through some similar prior works. Abdallah et al. (2015), in their study, showed that the increased MIC values to eravacycline up to 4 μg/mL corresponded to increase in the expression of AdeABC MDR pump. However, the upregulation does not always signify the resistance towards the induced antibiotic, as is the case, that no MDR specific resistance towards eravacycline has been reported in *Acinetobacter* [9]. The enzyme M1 family peptidase is present in many pathogens and is known to be a key enzyme for the survival in these organisms. It was notable that these enzymes were also upregulated in the antibiotic-treated ATCC 19606 strain, signifying the pressure of survival as induced by the presence of antibiotic. RND efflux pumps are a common mechanism involved in antibiotic resistance among *A. baumannii*, AdeB efflux pump is one of the upregulated proteins in the antibiotic-induced JU0126 strain. In *A. baumannii*, AdeABC is the first characterized efflux pump belonging to the RND superfamily*.* The operon codes for a major facilitator superfamily protein transporter protein AdeA, a multidrug transporter AdeB, and an outer membrane protein (OMP) AdeC. Eravacycline resistance in *A. baumannii* due to AdeABC efflux has not been reported before; hence, this upregulation can be attributed to the induction due to the antibiotic even though it is not a substrate for the pump. Antibiotic induced *adeB* efflux pump resistance has a major influence on the resistance status of *A. baumannii* [26]. The role of similar overexpression of *adeB* has been noted in some MDR isolates resistant to tigecycline in some of the previous research works [27]*.* Eravacycline induced overexpression of MacA efflux protein of the MacAB–TolC MDR pump expression was reported in *K. pneumoniae* emphasizing the role of efflux in eravacycline heteroresistance [28]. The next protein that was upregulated in the present study was an Omp38, which is a major porin protein from *A. baumannii*. OMPs are crucial proteins for antibiotic diffusion and membrane permeability; deficiency of which leads to increased susceptibility to antibiotic. Studies have shown increased production of OMPs, like OmpA38, CarO, OmpW, in the presence of tetracycline, suggesting that the overexpression relates to overcoming antibiotic stress [18]. *A. baumannii* is an organism that harbours multiple mechanisms for antibiotic resistance, and β-lactamases are a group that tackles the β-lactam drugs efficiently in these organisms. Class C extended-spectrum β-lactamase ADC-26 was seen upregulated in our study in JU0126 strain. The overexpression of ADC is reported to confer resistance to a range of β-lactam antibiotics making the infections caused by *A. baumannii* difficult to treat [29]. However, the overexpression in the eravacycline treated JU0126 could be due to a random antibiotic stress response because these β-lactamases does not have substrate specificity for a non-β-lactam drug.

Previous reports have demonstrated that OMVs isolated from antibiotics resistance strains help susceptible strains in transferring antibiotics resistance genes and proteins under antibiotic stress condition [21]. Carbapenem-resistant *A. baumannii* releases OMVs packed with carbapenem resistance-related genes and could undertake the horizontal transfer to carbapenem-susceptible *A*. *baumannii* [30]*.* In one study, OMVs from *E. coli* were found to seize antibiotics, such as colistin and degrade the antimicrobial peptides like melittin [31]. *Moraxella catarrhalis* and *Staphylococcus aureus* also releases OMVs, which carries β-lactamase helping the bacteria to survive in the presence of β-lactam antibiotics [32].

### Downregulated DEGs/proteins

The tetracycline group of antibiotics act by binding to ribosomal subunit 30S thereby blocking the aminoacyl-tRNA to bind to ribosomal acceptor site A; hence, inhibiting the protein synthesis [33]. It was reported by Vrentas et al., that the downregulation of RNA synthesis occurs as a result of protein synthesis inhibition [34]. In the present study, the transfer RNAs were downregulated in both the ATCC 19606 and JU0126 strain, which explains the adaptation of the bacterium to pressure, trying to keep the metabolic process minimal, similar reports on the reduced metabolism due to tigecycline induction was done by Liu et al. [35].

In our study, porin proteins were downregulated in the *A. baumannii* ATCC 19606 strain. The loss or downregulation of porins is a mechanism of resistance, wherein the bacteria reduce the cell permeability preventing antibiotic entry and decrease the susceptibility [36]. The presence of tetracycline leads to differential expression of porins proteins, either increase or decrease of which decides the permeability of the cell envelope. The downregulation of porins in *A. baumannii* in this present study corresponds to the previous claims on tetracycline leading to the downregulation of numerous porins in *Escherichia coli* strains [37].

### Subcellular localization of proteins from OMVs

The subcellular localization of the proteins expressed in the OMVs from both the eravacycline treated and untreated control strain was identified using pSORT-B 3.0. Their results give crucial information on the function of the protein, which can be compared with their expression pattern in the present study condition (upregulated or downregulated; antibiotic stress or antibiotic resistance). The pSORT-B categorizes the Gram-negative bacterial proteins into five major sites—the cytoplasm, the inner membrane, the periplasm, the outer membrane and the extracellular space [38].

The localization analysis in the current study was done to visualize the effect of antibiotic stress on the OMVs specifically focusing on their proteins and its functions. In both the ATCC 19606 and JU0126 strains, proteins with functions related to resistance and stress were predominant, like the outer membrane proteins, efflux pumps, β-lactamase associated resistance proteins, stress tolerance proteins and peptidases. It is known that the proteins from OMVs aid the invasiveness of the bacteria, and are enriched with toxins, bioactive and virulence proteins. OMVs are a key for bacterial survival with their role in bacterial self-defence, formation of biofilm, antibiotic resistance and host–immune response modulation [39, 40]. The exposure of cells to environmental contaminants (antibiotics) has potentially evolved bacterial OMVs, either with multidrug efflux pumps capabilities or with ability to catalyse degradation by sequestering antibiotics from the extracellular milieu [41, 42].

### DEGs pertaining to virulence factors

The proteins belonging to the type VI secretion systems (T6SSs), which are a new type among the bacterial secretion systems, were increased in their expression ranging from 18-fold to a minimum of 7-fold change in the *A. baumannii* JU0126 eravacycline treated strain compared with the control. T6SSs are associated with the pathogenicity in bacteria with the experimentally proven role in bacterial virulence [43]. T6SSs does a bacteriophage-like contractile injection of effector proteins puncturing into target cells and when they inject antibacterial toxins to competing for bacterial cells, they become ‘antibacterial’ T6SSs [44, 45]. Some of the other genes with differential expression in JU0126 strain alone were LuxR family transcriptional regulator (a crucial protein involved with quorum sensing) with a 7-fold change in expression. These proteins coordinate the expression of virulence factors, biosynthesis of antibiotics and transfer of plasmids, bioluminescence, and formation of biofilms [46].

The efficient induction of eravacycline resistance was evident with a 5-fold change in expression of the gene that encodes 50S ribosomal protein L2, which is an rRNA binding protein and helps in the interaction of 30S and 50S subunits in order for tRNA binding to happen and; hence, peptide bond formation [47]. This contradicts the action of eravacycline which negates the bacterial protein synthesis by binding to the 30S ribosomal subunit, stopping peptide chains formation [9]. In addition, along with ribosomal proteins, genes for the elongation factor G were increased in their expression by 4-fold change, EF-G has two roles; one, during the translocation and the other, in the ribosome disassembly [48]. Genes coding for the protein involved with cell metabolism, such as the d-alanyl-d-alanine carboxypeptidase, a serine peptidase was 4-fold differentially expressed. These proteins are associated with virulence in *Acinetobacter* sp. [49] and have been experimentally proven to be essential for intracellular replication in some bacteria [50].

The arginine succinyl transferase A (astA) enzyme [51] gene had an 8-fold increase in the expression of *A. baumannii* ATCC 19606 treated strain. AstA was found associated with healthcare-associated pathogen *A. baumannii* strains [52], and it has been attributed to the pathogenesis in other bacterial strains like uropathogenic *E. coli* (UPEC), and was reported as one of the virulence proteins in *E. coli* [53].

In both *A. baumannii* ATCC 19606 and JU0126 strains induced by eravacycline, the genes for type I secretion system and elongation factor TU had a positive log_2_-fold change, with a 3-fold change in the JU0126 strain. The type I secretion system helps in the secretion of proteins from cytoplasm to the extracellular region. They harbour a specific OMP for their export and one among the best-studied is TolC from *E. coli* [54]. Elongation factor TU is a GTPase also known to perform moonlighting functions on the surface of human pathogens acting as a multifunctional adhesin [55].

### DEGs as resistance determinants

Positive differential expression of many genes encoding resistance proteins was observed in both ATCC 19606 and JU0126 strains induced with eravacycline from the RNA sequence analysis. The efflux pumps and the ribosomal protection are the two main resistance mechanisms in *A. baumannii* to tetracycline class of drugs. In *A. baumannii* ATCC 19606, genes for all the major efflux pump family proteins had a positive differential expression, such as MFS, RND, multidrug and toxic compound extrusion (MATE) and ABC transporters. A 9-fold change in the expression of gene that codes for MFS transporter, many of which are involved in the drug efflux of antimicrobials, such as tetracyclines, fosfomycin, colistin and erythromycin [56] noted in the ATCC 19606 strain, whereas the JU0126 strain had a negative log_2_ change in the expression of this transporter. Tet efflux pumps are among the main types that come under MFS transporters, *tetA* gene codes for an efflux protein that confers resistance to tetracyclines. The *A. baumannii* has two pump proteins under MFS category (uses proton exchange for a tetracycline-cation), Tet(A) and Tet(B) [57].

A 7-fold increase in the expression of TetR/AcrR family transcriptional regulator gene was observed in the *A. baumannii* ATCC 19606, induced with eravacycline while their expression in JU0126 strain showed a negative log_2_-fold. The TetR family of regulators (TFR) comes under the signal transduction systems with the drug–efflux pump regulation as their functional role. The expression of *acrAB* efflux pump operon is repressed by the AcrR. TetR is a family of tetracycline transcriptional regulator that has a role in the transcriptional control. In the absence of tetracycline antibiotic, TetR binds to the Tet(A) gene to repress its expression. Tet(A) exports tetracycline from the cell before it can exert the protein synthesis inhibition [58].

The overproduction of RND pumps, such as AdeABC, AdeFGH, and AdeIJK is a major factor contributing to the resistance in *Acinetobacter* [59]. The gene for AdeB/AdeJ proteins had 3-fold differential expression in both the ATCC 19606 and JU0126 strains. AdeB is the multidrug transporter for the AdeABC tripartite efflux pump that expels out an array of antibiotics, such as aminoglycosides, β-lactams, chloramphenicol, erythromycin, and tetracyclines. This positive differential expression of AdeB can be correlated with the prior studies on which it was reported to be the most prevalent with increased expression among the MDR *A. baumannii* strains in Zhenjiang, China by Yang et al. [60]. Positive 6-fold differential expression of the multidrug efflux RND transporter permease subunit gene was noted in the ATCC 19606 strain, whereas a negative 3-fold decrease in the expression of the JU0126 strain. The ABC transporter ATP-binding protein gene expression was increased by 6-fold in the eravacycline induced ATCC19606 strain when compared with the uninduced strain; however, the MacB protein subunit was under-expressed with a negative 2-fold change in the same strain. The MacA–MacB–TolC is a three protein efflux system that expels out mainly macrolide class of antibiotics, and their expression may not be influenced in a large way by the eravacycline [61]. MATE family pumps are not much related to resistance towards the tetracycline class of drugs and basically confer resistance towards fluoroquinolones and imipenems [62]. However, there was a 7-fold change in the gene expression of MATE family pumps in the eravacycline induced *A. baumannii* ATCC 19606, but negative differential expression of negative 5-fold change in the JU0126 strain. Porins are the channel-forming protein that helps in the transport of molecules across the selectively permeable bacterial membrane bilayer. Mutations or changes in the porin proteins, such as loss or modification of the size of porin or lower expression result in the limited diffusion of β-lactams, fluoroquinolones, tetracycline and chloramphenicol [63]). Many of the genes coding for porins had both positive and negative-fold change and reduced differential expression among both the *A. baumannii* ATCC 19606 and JU0126 strains treated with eravacycline like the carbapenem susceptibility porin CarO (− 4- and − 0.2-fold change), OmpW family protein (− 3- and − 8-fold change), outer membrane porin OprD family (5- and − 4-fold change) and OmpA family protein (− 0.06 and 3-fold change). This reduction in the expression of these porins signifies their role in conferring resistance by decreasing the antibiotic entry into cell.

Although β-lactamase enzyme production is not related to the eravacycline resistance, few classes of β-lactamase were noted to have both positive and negative-fold change. The genes for enzymes MBL-fold metallohydrolase had 5-fold change and 4-fold change, OXA-51 family carbapenem-hydrolysing class-D β-lactamase OXA-259 with 1 and − 0.4-fold change and class C extended-spectrum β-lactamase ADC-26 with − 1-fold and 7-fold change for *A. baumannii* ATCC 19606 and JU0126 treated strains, respectively.

### OMVs proteins with function pertaining to stress and resistance

The proteins involved with virulence, stress response and antibiotic resistance expressed in the OMVs of eravacycline induced *A. baumannii* strains of ATCC 19606 and JU0126 were compared with the uninduced controls with respect to their log_2_-fold change (only proteins with more than 2 log_2_-fold change are mentioned below). Many proteins especially ribosomal proteins had more than 2 log_2_-fold change in the expression in both the ATCC 19606 and clinical strain JU0126 and apart from that chaperons, OMP and resistance-conferring proteins were observed. Prior studies have also reported many OMP [31, 64] and resistance-conferring proteins expressed in OMVs of antibiotic-treated strains, our study identified many OMP and antibiotic resistance-related proteins from both *A. baumannii* ATCC 19606 and JU0126. In the ATCC 19606 strain, highest log_2_-fold change was for OmpA family protein (5.66), followed by Omp38 (4.43), β-lactamase (3.40), OprD family (2.91) and putative acriflavine resistance protein A (2.30). Other proteins pertaining to virulence, stress and bacterial survival with more than 2 log_2_-fold change were copper-exporting ATPase (9.65) which is a copper tolerance protein, toluene tolerance protein Ttg2D (8.87), TonB-dependent siderophore receptor (6.69), 50S ribosomal proteins L14, L6, L4, L19, L16, L29 and L2 (log_2_-fold change range from 2 to 6), 30S ribosomal proteins S11, S3 and S7 (log_2_-fold change 3–4), peptidases S41 family (6.53), peptidoglycan-associated protein (6.0), type IV pilus biogenesis/stability protein PilW (4.94), type VI secretion protein, EvpB/VC_A0108 family (3.15), translation initiation factor IF-3 (4.12), TolB belonging to the Tol–Pal peptidoglycan-associated lipoprotein system protein (3.34), chaperone protein HscA homolog that belongs to the heat shock protein 70 family (2.75) and vacJ-like lipoprotein. There were just two proteins associated with resistance showing more than 2 log_2_-fold change in the OMV proteome of *A baumannii* JU0126 strain, β-lactamase protein, and major facilitator family transporter. However, many stress response proteins, virulence, and survival proteins were expressed with more than 2 log_2_-fold change in JU0126. The same as *A. baumannii* ATCC 19606, ribosomal protein abundance was very significantly high noting that the strains were induced resistance to eravacycline. 30S ribosomal proteins S5, S4, S2, S3, S9 ranged from 2 log_2_-fold change to 9 log_2_-fold change and the 50S ribosomal proteins L4, L6, L2, L1, L18, L16, L28, L10 with log_2_-fold ranging between 4 and 8. Other proteins like toluene tolerance protein Ttg2D, Tol–Pal system protein TolB, gamma-glutamyl transferase, acetyl-CoA C-acetyltransferase, transcription termination factor Rho, YqaJ viral recombinase family protein, signal recognition particle protein, TonB-dependent siderophore receptor, and peptidases M48, S41 were expressed with more than 2 log_2_-fold change in the eravacycline induced strains.

### Inconsistency in the expression patterns of OMVs proteins in comparison to the bacterial whole gene expression profiles

The overall results from the comparison of the two expression profiles, the protein, and the RNA were with a very low correlation coefficient. Some of the ribosomal proteins were upregulated in both RNA and OMV proteome expression profiles. The expression of ribosomal proteins in the OMV proteome can be supported by reports on the presence of RNAs and the proteins involved in their synthesis. Sjöström et al. (2015) reported for the first time that RNAs were involved with bacterial OMVs [65]. Other proteins with a correlation between mRNA and protein expression include dihydrolipoamide acetyltransferase, DUF4142 domain-containing protein, class C extended-spectrum β-lactamase ADC-26 and a hypothetical protein. In the strain *A. baumannii* ATCC 19606, although many ribosomal proteins showed upregulation in their expression, linear correlation of both mRNA and protein expression was seen only in, copper-translocating P-type ATPase, methylmalonate-semialdehyde dehydrogenase (CoA acylating), adenosine deaminase and gamma-glutamyltransferase family g-protein. The low correlation of the mRNA and protein components based on the log_2_-fold change comparison suggests that proteins in OMVs are selectively enriched, transported from the bacterial cell and/or due to wide range of regulatory mechanisms involved in the post-transcriptional level [66]. A poor correlation of similar comparison was reported by Yun et al. (2018) in their study of proteins in OMVs and protein fractions from bacterial cell membranes. They have mentioned the reason to be that proteins in the OMVs are differentially selected and sorted from the host bacteria.

### Enriched biological pathways

PPI networks from commonly expressed gene/proteins from both transcriptome and OMVs proteome of ATCC 19606 and JU0126 strains were constructed. Pathways that were found enriched were significantly ironically related to transcription and RNA synthesis, owing to the fact that the bacterium was grown in an eravacycline stressed environment and the subsequent induced resistance.

## Conclusion

The transcriptome of the whole cell and OMVs proteome abundance was studied for two *A. baumannii* strains, one an ATCC 19606 and a clinical isolate JU0126 strain in an eravacycline induced antibiotic resistant condition. From the whole-cell RNA sequence analysis, different virulence factors, resistance genes were upregulated, whereas the OMVs proteome was enriched with more proteins essential for bacterial stress and survival. The network interactions and respective MCODE cluster information clearly correlate with the study growth conditions with high eravacycline concentrations and the induced resistance towards the antibiotic in the bacterium. The observation from this study is that eravacycline greatly upregulates the resistance-conferring genes in the whole cell, whereas not many resistance-related effects were seen in the OMVs proteome. This work focused on the differential proteome of OMVs and their possible influence in the induced resistance to eravacycline; however, it was found from the outcome of the results that OMVs rather support the bacterial survival with its stress proteins, chaperones and proteases more than the resistance-conferring abilities. OMVs are essential although not alone, but in close unison with the bacterial cellular factors for the resistance and sustenance in the lethal eravacycline concentrations.

## Methods

### Bacterial strains

*A. baumannii* JU0126 clinical strain was a previously characterized MDR clinical isolate obtained from a patient diagnosed with fever in Jiangbin Hospital, Zhenjiang, Jiangsu Province, China. The strain was resistant to tigecycline but susceptible to eravacycline. *A. baumannii* ATCC 19606 was used as a reference strain. Further, the minimal inhibitory concentration of eravacycline antibiotic was ascertained for both the strains.

### Induction of eravacycline resistance

A single colony of both ATCC 19606 and JU0126 strain were inoculated into the cation adjusted Mueller–Hinton broth (CAMHB) containing sub-MIC concentration of eravacycline incubated at 37 °C at 250 rpm overnight. On day 3, 0.1 mL culture suspension was transferred into the freshly prepared CAMHB (10 mL) with next higher concentration of eravacycline and incubated at 37 °C at 250 rpm overnight. This passage was continued until the maximum concentration above the MIC of eravacycline was achieved, that the strains were able to resist and grow in the same incubation conditions [67, 68]. The growth suspension from the sub- MIC concentration (after in vitro induction of resistance) was platted on MHA plate (containing the final eravacycline concentration used for induction) and a single colony from the MH plate was taken for total RNA isolation (performed as duplicates).

### RNA sequencing

The quality and quantity of the total RNA from both *A. baumannii* ATCC 19606 and JU0126 strains were assessed using the NanoPhotometer® spectrophotometer (IMPLEN, CA, United States) and Qubit® RNA Assay Kit in Qubit® 2.0 Flurometer (Life Technologies, CA, United States), respectively followed by RNA sequence analysis. The RNA integrity number (RIN) was calculated using the RNA Nano 6000 Assay Kit of the Bioanalyzer 2100 System (Agilent Technologies, CA, United States). The RNA-sequencing library was constructed using NEBNext® Ultra™ Directional RNA Library Prep Kit for Illumina® (NEB, United States) as per the manufacturer’s instructions.

The purification of library fragments was done using AMPure XP system (Beckman-Coulter, Beverly, United States), and 3 μL USER Enzyme (NEB, United States) was used with size-selected, adaptor-ligated cDNA to get 150–200 bp sized cDNA. Phusion high-fidelity DNA polymerase, Universal PCR primers, and Index (X) primers were used for the PCR; and AMPure XP system was used to purify the PCR products, and the quality was thus assessed using Agilent Bioanalyzer 2100 system. Clusters were generated using a cBot Cluster Generation System using TruSeq PE Cluster Kit v3-cBot-HS (Illumia), and the library preparations were sequenced using Illumina Hiseq platform.

### Analysis of the RNA-sequence data

The sequenced libraries were mapped against predicted transcripts from the *A. baumannii* ATCC 19606 genome using TopHat v2.0.4. HTSeq v0.6.1 was used to count the read numbers mapped to each gene, an abundance of transcript (FPKM, fragments per kilobase of exon per million fragments mapped) and significant changes in transcript expression were estimated using Cufflinks v2.0.2. The read counts for the sequenced libraries were adjusted using edgeR program package through one scaling normalized factor, and this was followed by differential expression analysis of two conditions/groups (two biological replicates per condition) using the DESeq R package (1.18.0). GO seq R package was used for Gene Ontology (GO) enrichment analysis of differentially expressed genes (DEGs), and the statistical enrichment was done using STRAP software [22].

### Reverse transcriptase-quantitative PCR

Gene expression was analysed using a previously described method [69]. Briefly, total RNA was isolated from 1 × 10^9^*A. baumannii* cells. After the treatment with DNase, RNA samples were taken for cDNA synthesis. The template cDNA was diluted to 1:100, and 2.5 μL of which was added to SYBR green PCR master mix for each reaction and Applied Biosystems™ StepOne™ Real-Time PCR was used for the analysis. Both internal forward and reverse primers were designed using IDA website (Additional file 1). The experiments were repeated in independent duplicates. Normalization to the *gyrB* genes facilitated the calculation of the fold changes using the threshold cycle (Ct).

### Purification of OMVs

OMVs of both *A. baumannii* ATCC 19606 and JU0126 were prepared from previously described methods [70, 71]. In brief, the eravacycline treated and untreated *Acinetobacter baumannii* were grown in 500 ml Luria Bertani (LB) broth until the OD at 600 nm reached 1.0 at 37 °C in incubator shaker with (sub-MIC concentration achieved upon induction of resistance) eravacycline, that is 8 μg/ml for *A. baumannii* ATCC 19606 strain and 32 μg/ml for *A. baumannii* JU0126 strain) and without eravacycline. Culture suspension was then centrifuged at 6000 *g* at 4 °C for 15 min to remove bacterial cells. The supernatants were filtered through vacuum filter (0.22 μm size) to remove the cell debris. And filtered samples were concentrated using 100 KDa Merck ultrafiltration tube. The samples were taken for ultracentrifugation at 150,000 *g* at 4 °C for 3 h, pellets were resuspended in phosphate buffer saline and protein concentration was determined using modified BCA assay (Thermo Scientific). The OMVs were initially fixated, and the ultrathin sections were stained using 3% uranyl acetate negative staining technique and imaged using Transmission Electron Microscope (Philips). The OMVs were stored at − 80 °C after sterility check for further use.

### LC-MS/MS analysis of OMVs

OMVs proteins were identified by one-dimensional electrophoresis–liquid chromatography-tandem mass spectrometry using nano-LC LTQ-Orbitrap Mass Spectrometer, Thermo Fisher Scientific, Bremen, Germany. OMVs protein was trypsin digested, and each fraction was reconstituted in HPLC grade 5% acetonitrile and 0.1% formic acid (solvent A) and then loaded on to the nano HPLC column. A gradient was formed, and the peptides were eluted with increasing concentration of 98% acetonitrile and 1% formic acid (solvent B). The eluted peptides were detected in the ESI mass spectrometer and produced a tandem mass spectrum of specific fragment ions for each peptide [72].

### Identification and quantification of proteins from OMVs

LC-MS/MS raw data were used to identify the peptides/proteins from OMVs using MaxQuant (version 1.6.3.4) with match between runs, matching time window of 2 min. The search parameters are as follows: enzymes specify—trypsin; variable modification—oxidation of methionine (15.995 Da); fixed modification—carbamidomethylation of cysteine (57.021 Da); two missed cleaves; precursor ions tolerance—20 ppm and fragment ions tolerance—4.5 ppm. Reference proteome of *A. baumannii* ATCC 19606 was retrieved from Uniprot database. Contaminant sequences were used for search and seven amino acids were set as the minimum length of peptide for analysis. The first majority proteins ID were selected and used for further analysis. Uniprot database and primary location were used to generate the protein location. Using DAVID web tool (https://david.ncifcrf.gov/), biological terms were generated and proteins identified from MS analysis were annotated for subcellular localization using pSORTb version 3.0.2 [38].

### Protein-protein interaction network (PPI) analysis for the gene/protein

PPIs for *A. baumannii* ATCC 19606 and JU0126 strains were obtained from string database [73]. Potential PPIs were constructed for the common gene/protein from transcriptome and proteome analysis, respectively, using Cytoscape v3.7.1. The molecular complex detection (MCODE) algorithm was used to find highly interconnected subgraphs to find densely connected regions in the PPI network [74]. Using MCODE plug-in highly interconnected nodes (*n* > 10) were identified and clustered as subnetwork. Further, identified clusters from MCODE were used to find function enrichment using ClueGO/CluePediaplug-in of Cytoscape software [75, 76].

## Supplementary information

**Additional file 1.** List of primers used for the RT PCR analysis.

**Additional file 2.**Gene expression data from the whole cell transcriptome analysis of *A. baumannii* ATCC19606 and JU0126 strain, showing mRNA expression levels of eravacycline treated versus control.

**Additional file 3.**DEGs of eravacycline treated and control strains of *A. baumannii* ATCC19606 and JU0126.

**Additional file 4.**LC/MS–MS proteomic analysis of OMVs from eravacycline treated and control strains of *A. baumannii* ATCC19606 and JU0126.

**Additional file 5.**Comparative analysis of transcriptome and OMVs proteome of eravacycline treated and control strains of *A. baumannii* ATCC19606 and JU0126.

**Additional file 6.**The PPI network of genes/proteins expressed commonly in transcriptome and OMVs proteome from *A. baumannii* ATCC 19606 and JU0126 strains.

**Additional file 7.**Subnetworks identified using MCODE plug-in in the PPI network of *A. baumannii* ATCC 19606 and JU0126strain.

**Additional file 8.**Schematic representation of MCODE clusters of *A. baumannii* ATCC 19606 and JU0126 strains.

## Data Availability

All data generated during this study are included in this published article and its additional files.

## Abbreviations

*A. baumannii*: *Acinetobacter baumannii*
ABC: ATP-binding cassette superfamily
astA: Arginine succinyl transferase A
ATCC: American Type Culture Collection
CAMHB: Cation adjusted Mueller–Hinton broth
DEGs: Differentially expressed genes
JU0126: Jiangsu university strain No. 0126
KEGG: Kyoto encyclopedia of genes and genomes
LB: Luria Bertani
LC-MS/MS: Liquid Chromatography with tandem mass spectrometry
MATE: Multi antimicrobial extrusion protein family
MCODE: Molecular Complex Detection
MDR: Multidrug resistance
MFS: Major facilitator superfamily
OMP: Outer membrane protein
OMVs: Outer membrane vesicles
PPI networks: Protein-protein interaction network
qRT-PCR: Quantitative reverse transcriptase-polymerase chain reaction
RND: Resistance-nodulation-cell division superfamily
STRAP: Software Tool for Rapid Annotation of Proteins
UPEC: Uropathogenic *E. coli*
VAP: Ventilator-associated pneumonia
