# Supplementary material for: Integrative analysis of outer membrane vesicles proteomics and whole-cell transcriptome analysis of eravacycline induced Acinetobacter baumannii strains
Source: BMC Microbiol. 2020 Feb 11;20:31. doi: 10.1186/s12866-020-1722-1 (PMC7014627; doi:10.1186/s12866-020-1722-1)
Supplement: Supplementary file 1 — Additional file 1. List of primers used for the RT PCR analysis. [file 12866_2020_1722_MOESM1_ESM.docx]

**Additional file 1:** List of primers used for the RT PCR analysis.

| **S.no** | **DEG** | **Genes** | **Primer** | **Sequences** |
| --- | --- | --- | --- | --- |
| 1 |  | gyrB | Forward | CGAGGGTGACTCAGCGGGTG |
|  |  |  | Reverse | GCGCACGCTCAACGTTCAGG |
| 2 | 126 Upregulated | Class C extended spectrum | Forward | CCTCAATTTATGCGGGCAATAC |
|  |  |  | Reverse | GCCATACCTGGCACATCATA |
| 3 |  | Multidrug efflux RND | Forward | CCATATTTGCGTGGGTGATTG |
|  |  |  | Reverse | GTGGTGCAATCGTTGGATATTG |
| 4 |  | OmpA family protein | Forward | GCGAATACACGACGGTTCATA |
|  |  |  | Reverse | TGATGCTTCTCGTTTGTCTACTC |
| 5 | 126 Downregulated | Transfer-messenger RNA | Forward | GCCGGTGATGAAACTCATAGA |
|  |  |  | Reverse | GTTTCGTCGTTTGCGACTATTT |
| 6 |  | Iron-containing alcohol | Forward | GGAACCTAACCCAACCGATATT |
|  |  |  | Reverse | ACCGCCTAACGACACAATAAA |
| 7 |  | Aldehyde dehydrogenase | Forward | GAAGTCTGGTATTGGTCGTGAA |
|  |  |  | Reverse | GCCCATTGGTTTGGTTGAATAA |
| 8 | ATCC 19606 Upregulated | Multidrug efflux | Forward | GGGTTGGTGAAGTCGGATTAT |
|  |  |  | Reverse | CACAATTTCAGTGGCTGTAAGG |
| 9 |  | MFS transporter | Forward | CACAAGGTATTGAGGTGGGTAG |
|  |  |  | Reverse | AGCACCATAAGCGAGGAATAAA |
| 10 |  | M1 family peptidase | Forward | GCTCAGATGTTTGTTGGGTATTG |
|  |  |  | Reverse | GCCAGTCATTTCCGTCATTTG |
| 11 | ATCC 19606 Downregulated | Putative porin | Forward | TACGACGAGAAAGACGGTAAAG |
|  |  |  | Reverse | GTCACCAAACGCACCATAAG |
| 12 |  | Transfer-messenger RNA | Forward | GCCGGTGATGAAACTCATAGA |
|  |  |  | Reverse | GTTTCGTCGTTTGCGACTATTT |
| 13 |  | Trifunctional transcriptional regulator | Forward | GGGAAGTTCCATGGGCTAATAC |
|  |  |  | Reverse | AGTTCGTGAGGCTGTAGATTTC |

DEGs: differentially expressed genes.
