# Supplementary material for: Integrative analysis of outer membrane vesicles proteomics and whole-cell transcriptome analysis of eravacycline induced Acinetobacter baumannii strains
Source: BMC Microbiol. 2020 Feb 11;20:31. doi: 10.1186/s12866-020-1722-1 (PMC7014627; doi:10.1186/s12866-020-1722-1)
Supplement: Supplementary file 6 — Additional file 6.The PPI network of genes/proteins expressed commonly in transcriptome and OMVs proteome from A. baumannii ATCC 19606 and JU0126 strains. [file 12866_2020_1722_MOESM6_ESM.pdf]

.

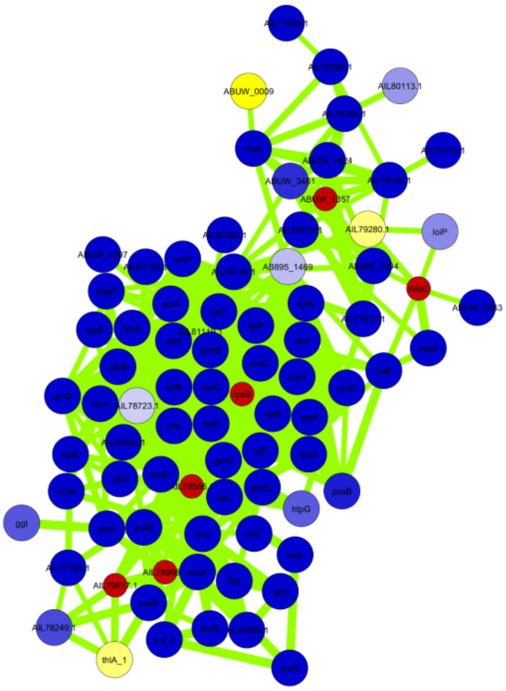

A

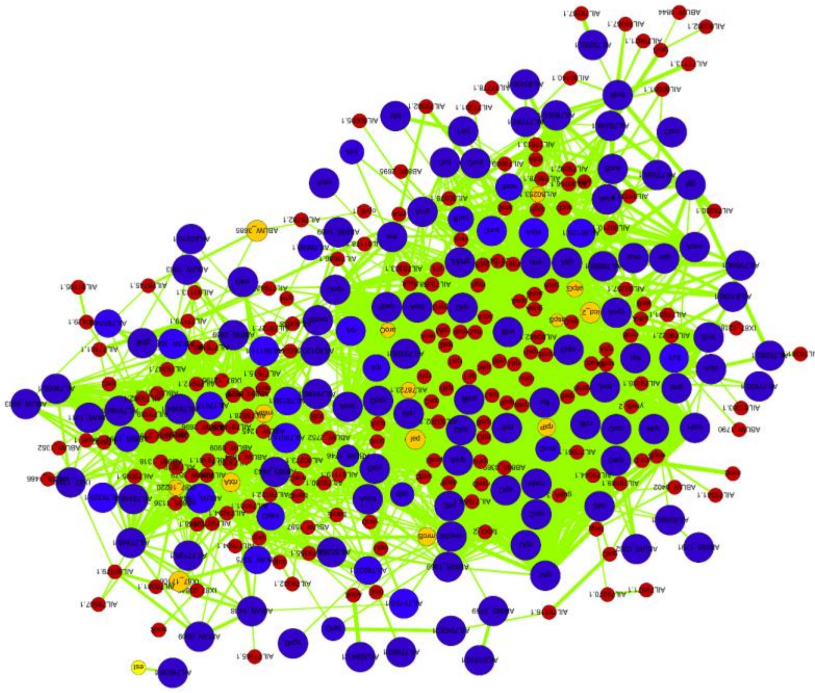

B

Additional file 6 | A, **B**: The PPI network of genes/proteins expressed commonly in transcriptome and OMVs proteome from *A. baumannii* ATCC 19606 and JU0126 strains. The blue and yellow nodes represent commonly identified genes/proteins from both mRNA and OMVs proteome and red nodes represent neighboring proteins. The size of the blue and yellow nodes represented in *p*-value and edge size of the PPI network were represented in combine score.
