# Supplementary material for: Integrative analysis of outer membrane vesicles proteomics and whole-cell transcriptome analysis of eravacycline induced Acinetobacter baumannii strains
Source: BMC Microbiol. 2020 Feb 11;20:31. doi: 10.1186/s12866-020-1722-1 (PMC7014627; doi:10.1186/s12866-020-1722-1)
Supplement: Supplementary file 7 — Additional file 7.Subnetworks identified using MCODE plug-in in the PPI network of A. baumannii ATCC 19606 and JU0126strain. [file 12866_2020_1722_MOESM7_ESM.docx]

**Additional file 7 |** Subnetworks identified using MCODE plug-in in the PPI network of *A. baumannii* ATCC 19606 and JU0126strain.

| Sub-network | MCODE score | Number of proteins (Nodes) | Number of interactions (Edges) | GO term Functions |
| --- | --- | --- | --- | --- |
| **ATCC** | | | |  |
| CLUSTER 1 | 51.321 | 54 | 1360 | ribosomal proteins, RNA polymerases, intracellular organelles, tRNA binding, regulation of translation |
| CLUSTER 2 | 10.600 | 11 | 53 | cell envelope organization |
| CLUSTER 3 | 9.882 | 18 | 84 | response to toxic substance |
| CLUSTER 4 | 7.200 | 11 | 36 | Proteins of unknown functions |
| **JU0126** | | | |  |
| CLUSTER 1 | 23.333 | 25 | 280 | Ribosomal subunit, RNA polymerase, cellular macromolecular biosynthesis, cellular nitrogen compound biosynthetic process |
