# Supplementary material for: Integrative analysis of outer membrane vesicles proteomics and whole-cell transcriptome analysis of eravacycline induced Acinetobacter baumannii strains
Source: BMC Microbiol. 2020 Feb 11;20:31. doi: 10.1186/s12866-020-1722-1 (PMC7014627; doi:10.1186/s12866-020-1722-1)
Supplement: Supplementary file 8 — Additional file 8.Schematic representation of MCODE clusters of A. baumannii ATCC 19606 and JU0126 strains. [file 12866_2020_1722_MOESM8_ESM.docx]

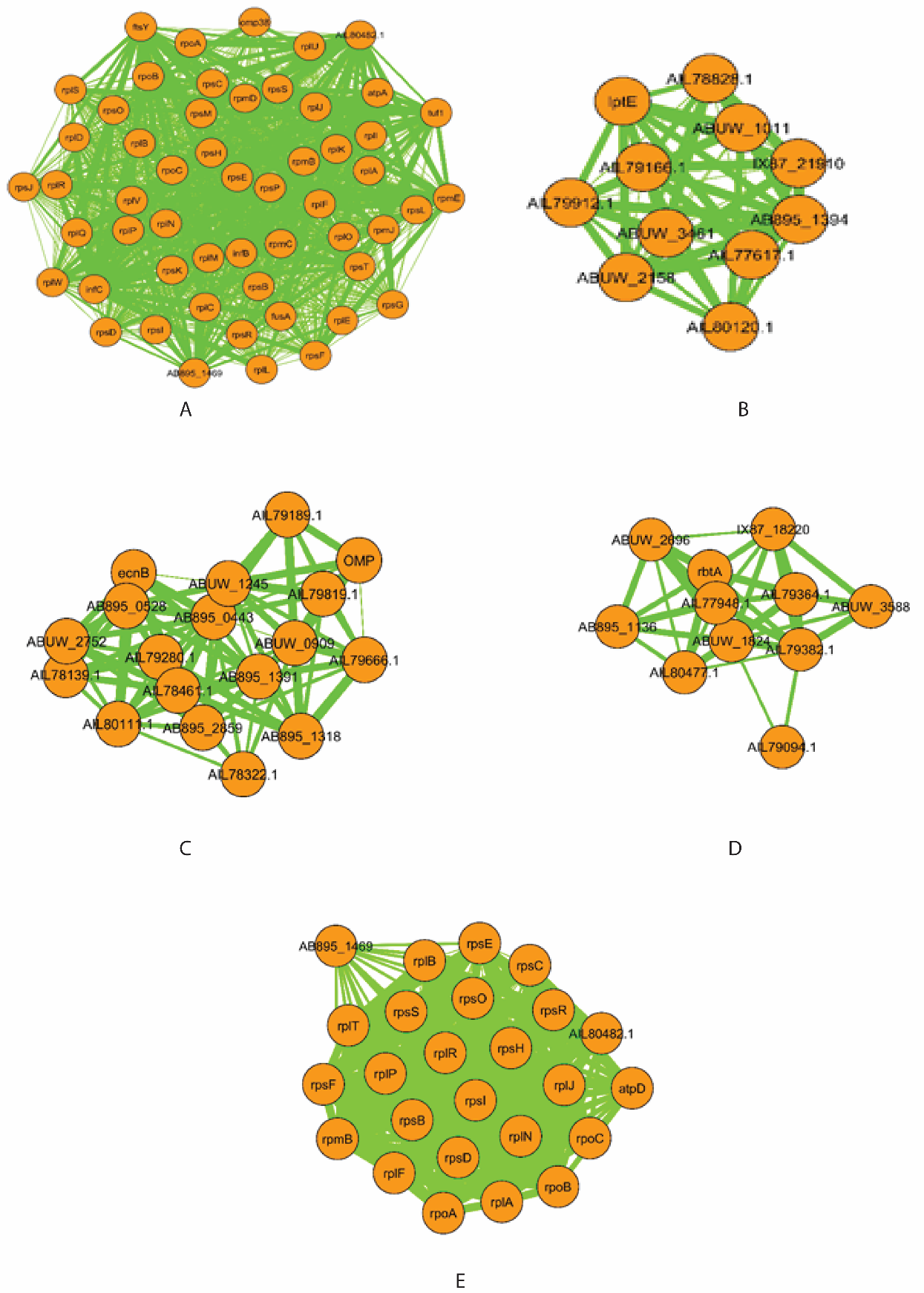


**Additional file 8** : Schematic representation of MCODE clusters of *A. baumannii* ATCC 19606 (A-D) and JU0126 (E) strains. Nodes colors represent in orange color, and the size of edges was represented in combine score in PPI network.
